# 一个遗传性凝血因子Ⅴ缺陷症家系的分子致病机制研究

**DOI:** 10.3760/cma.j.cn121090-20240424-00161-1

**Published:** 2024-12

**Authors:** 朗译 秦, 怡 陈, 玲玲 侯, 艳慧 金, 一凡 芦, 柯 张, 明山 王

**Affiliations:** 1 温州医科大学附属第一医院医学检验中心，浙江省检验诊断及转化研究重点实验室，温州 325015 Department of Clinical Laboratory, Key Laboratory of Clinical Laboratory Diagnosis and Translational Research of Zhejiang Province, the First Affiliated Hospital of Wenzhou Medical University, Wenzhou 325015, China; 2 温州医科大学附属第一医院血液科，温州 325015 Department of Hematology, the First Affiliated Hospital of Wenzhou Medical University, Wenzhou 325015, China

**Keywords:** 凝血因子Ⅴ缺陷症, 遗传性疾病, 纯合子, 近亲结婚, Coagulation factor Ⅴ deficiency, Genetic disease, Homozygous mutation, Consanguineous marriage

## Abstract

**目的:**

探讨一个姑表近亲结婚导致的遗传性凝血因子Ⅴ（FⅤ）缺陷症家系分子致病机制。

**方法:**

检测家系成员（3代7人）凝血参数和先证者及其父亲的凝血活酶生成量。用Sanger测序法分析先证者F5基因全部外显子，发现突变位点后反向测序证实，再检测家系成员相应位点。运用多个在线软件预测突变位点的保守性及致病性。依据美国医学遗传学与基因组学学会（ACMG）指南标准化评估该突变点位的致病性。

**结果:**

先证者凝血酶原时间（PT）、活化部分凝血酶原时间（APTT）分别为52.2 s、108.3 s，FⅤ活性（FⅤ∶C）、FⅤ抗原（FⅤ∶Ag）严重降低（分别为2％、4％），诊断为Ⅰ型FⅤ缺陷症。其父亲、母亲、外祖父的PT和APTT稍高于参考值，FⅤ∶C和FⅤ∶Ag为参考值的50％左右。血浆凝血活酶生成试验显示先证者及其父亲的凝血活酶生成量低于健康对照，先证者凝血活酶生成能力受损更为严重。直接测序结果显示先证者F5基因第15号外显子存在c.5128T>C（p.Trp1682Arg）纯合错义突变。先证者外祖父、父亲、母亲均为c.5128T>C的杂合子。保守性分析显示p.Trp1682为高度保守位点，Mutation taster、SIFT、REVEL、PolyPhen-2、CADD在线软件分析均提示该突变具有致病性。根据ACMG指南，新突变c.5128T>C为可能致病突变（PM2+PM3+PP1+PP3+PP4）。对比突变前后蛋白质模型发现突变后减少了一个苯环及一条氢键，使FⅤ蛋白局部结构发生改变。

**结论:**

F5基因第15外显子c.5128T>C（p.Trp1682Arg）错义突变初步考虑是该FⅤ缺陷症家系的遗传学病因，此突变为国际首次报道。

## Abstract (en)

**Objective:**

This study aimed to primarily discuss the pathogenesis of hereditary coagulation factor Ⅴ（FⅤ）deficiency in a family with a consanguineous cousin marriage.

**Methods:**

The coagulation indices of the pedigree（three generations with seven individuals）and the thrombin levels of the proband and his father were assessed. All exons of the F5 gene were analyzed with Sanger sequencing, and a new mutation was confirmed with reverse sequencing. The corresponding sites of the family members were then determined. A set of online software was utilized to predict the conservation and pathogenicity of the mutation site. The pathogenicity of this mutation site was evaluated according to the American College of Medical Genetics and Genomics（ACMG）guidelines.

**Results:**

The prothrombin time（PT）and activated partial thromboplastin time（APTT）of the proband were 52.2 s and 108.3 s, respectively. FⅤactivity（FⅤ∶ C）and FⅤ antigen（FⅤ∶Ag）were greatly decreased by 2% and 4%, respectively. The problem was diagnosed as typeⅠ FⅤ deficiency. PT and APTT of the proband's father, mother, and grandfather were slightly higher than the upper limit of the reference range, and FⅤ∶C and FⅤ∶Ag were approximately 50% of normal. The thromboplastin generation assay revealed that the amount of thromboplastin produced by the proband and his father was lower than that of the healthy controls and that the proband's ability to produce thromboplastin was more severely impaired. Sequencing analysis revealed that the proband demonstrated a homozygous missense mutation of c.5128T > C（p.Trp1682Arg）in exon 15 of the F5 gene. The grandfather, father, and mother of the proband were all heterozygous for c.5128 T > C. Conservative analysis revealed that p.Trp1682 was a highly conserved site in the homozygous species, and five online software programs, including Mutation Taster, SIFT, REVEL, PolyPhen-2, and CADD, indicated that the mutation was pathogenic. The ACMG guidelines recommend that the new mutation c.5128 T > C is a possible pathogenic mutation（PM2 + PM3 + PP1 + PP3 + PP4）. The comparison of the protein models before and after the mutation revealed that the benzene ring and the hydrogen bond were reduced after the mutation, which changed the local structure of the FⅤ protein.

**Conclusion:**

The missense mutation c.5128T > C（p. Trp1682Arg）in exon 15 of the F5 gene was initially considered the genetic cause of the FⅤ deficiency family. This mutation is the first report globally, which further enriches the gene-phenotype spectrum of FⅤ deficiency.

凝血因子Ⅴ（FⅤ）是一种主要由肝脏合成、分子量为330×10^3^的单链糖蛋白，约75％的FⅤ存在于血浆中，其余25％储存在血小板的α颗粒中[1]。编码FⅤ的F5基因位于1号染色体长臂（1q24），全长约80 kb，包含25个外显子，共编码2 224个氨基酸[2]。遗传性FⅤ缺陷症是一种罕见的常染色体隐形遗传缺陷症，在人群中发病率约为百万分之一[3]，主要由F5基因突变导致，于1947年由Owren首次报道[4]。FⅤ缺陷症可分为Ⅰ型和Ⅱ型，前者FⅤ活性（FⅤ∶C）及抗原（FⅤ∶Ag）均降低，后者FⅤ∶C降低而FⅤ∶Ag正常[5]。本研究对1个姑表近亲婚配导致的遗传性FⅤ缺陷症家系进行分析并初步探讨该家系的分子发病机制。

## 对象与方法

1. 家系资料：先证者，女，5岁，维吾尔族，新疆柯坪县人，因“无明显诱因双侧鼻出血2天”就诊。凝血常规检测发现凝血酶原时间（PT）和活化部分凝血活酶时间（APTT）分别为52.2 s（参考值范围12.5～14.5 s）、108.3 s（参考值范围29.0～43.0 s），FⅤ∶C、FⅤ∶Ag分别为2％、4％，其他凝血指标无明显异常，初步诊断为Ⅰ型FⅤ缺陷症。该家系共3代7名成员，先证者祖母（Ⅱ_2_）和外祖父（Ⅱ_3_）为姐弟，其父母为姑表近亲结婚，家系成员均无自发出血史。家系图见图1。

**图1:**
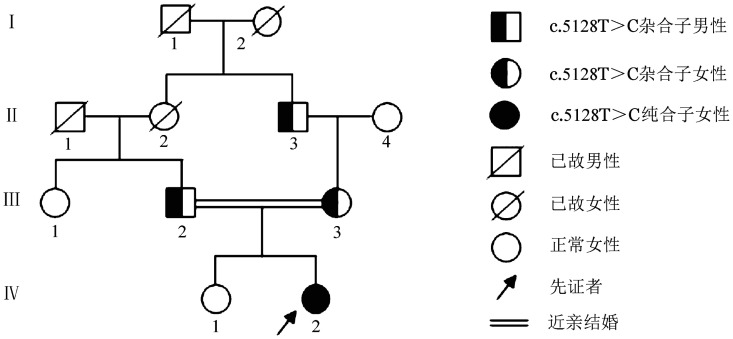
遗传性凝血因子Ⅴ缺陷症家系图

选取100名健康体检者作为对照组，其中男51名，女49名，中位年龄33（18～60）岁，均无肝肾功能异常，无自发出血史，无口服抗凝药史。本研究通过温州医科大学附属第一医院伦理审查（KY2022-R193），所有受试者均签署知情同意书。

2. 标本采集与处理：采集所有研究对象静脉血，于0.109 mol/L枸橼酸钠溶液抗凝管中混匀，×1 500 *g*离心15 min，取上层乏血小板血浆用于凝血检测及先证者血浆凝血活酶生成试验，下层血细胞用于DNA提取。

3. 凝血指标检测：PT、APTT、纤维蛋白原（FIB）、凝血因子Ⅱ活性（FⅡ∶C）、FⅤ∶C、凝血因子Ⅶ活性（FⅦ∶C）、凝血因子Ⅷ活性（FⅧ∶C）、凝血因子Ⅹ活性（FⅩ∶C）和狼疮抗凝物（LA）等指标在STA-R-Max全自动血凝仪上进行检测（法国Stago公司及配套试剂）；采用酶联免疫吸附测定FⅤ∶Ag，试剂盒购自温州长风生物技术有限公司。所有操作步骤均按照说明书进行。

4. 凝血活酶生成试验：使用Fluoroskan Ascent FL读数仪（美国Thermo Fisher公司）检测凝血活酶生成延迟时间（Lag time）、峰高（Peak）、到达峰值时间（ttPeak）和内源性凝血活酶生成潜力（ETP）4个参数，来评估先证者及其家系成员血浆FⅤ促凝能力。

5. F5基因测序及分析：采用DNA提取试剂盒（购自北京天根生化科技有限公司）提取先证者全血基因组DNA。于ABI 7500实时定量PCR仪（美国Thermo Fisher公司）上扩增F5基因所有外显子，引物及PCR扩增条件见文献[6]。扩增产物送上海赛恒生物科技有限公司直接测序。使用Chromas v2.23软件将测序结果与野生型F5基因序列进行比对，确定突变位点后，行反向测序加以证实，再检测其余家系成员相应外显子片段并进行测序分析。

6. 突变位点保守性及致病性分析：用ClustalⅩ-2.1-win软件将突变位点与NCBI（https://www.ncbi.nlm.nih.gov/guide/homology/）上其余8个同源物种：褐家鼠、小家鼠、黑猩猩、猕猴、牛、家犬、原鸡、斑马鱼进行F5基因氨基酸序列比对。使用以下在线生物信息学软件预测该突变位点致病性，包括Mutation taster（https://www.mutationtaster.org/）、SIFT（https://sift.bii.a-star.edu.sg/）、REVEL（https://genome.ucsc.edu/cgi-bin/hgTrackUi?db=hg19&g=revel）、PolyPhen-2（http://genetics.bwh.harvard.edu/pph2/index.shtml）和CADD（https://cadd.gs.washington.edu/）。

7. 蛋白质模型分析：以UniProt（https://www.uniprot.org/）中提供的蛋白结构（PDB ID:7KVE）为模型，在PyMOLWin-2.3软件中对比突变前后氨基酸空间结构的改变，分析分子间作用力的改变，推测该位点突变对蛋白功能影响。

## 结果

1. 凝血表型结果检测：先证者PT、APTT分别为52.2 s、108.3 s，FⅤ∶C、FⅤ∶Ag分别为2％、4％；家系成员中，其父亲、母亲、外祖父的PT和APTT稍高于参考值范围上限，FⅤ∶C和FⅤ∶Ag均明显低于参考值范围下限；家系其他成员的凝血指标均在参考值范围内。详见表1。

**表1:** 遗传性凝血因子Ⅴ缺陷症家系成员凝血检测结果

家系成员	PT（s）	APTT（s）	FIB（g/L）	FⅡ∶C（%）	FⅦ∶C（%）	FⅧ∶C（%）	FⅩ∶C（%）	FⅤ∶C（%）	FⅤ∶Ag（%）
外祖父（Ⅱ_3_）	17.8	49.0	3.84	95	99	123	90	55	53
外祖母（Ⅱ_4_）	13.7	42.8	2.96	94	100	118	92	93	90
父亲（Ⅲ_2_）	16.6	48.4	3.20	101	97	112	86	53	52
母亲（Ⅲ_3_）	16.3	43.5	2.45	98	87	101	102	46	54
姑母（Ⅲ_1_）	13.1	43.6	2.40	103	97	109	88	90	88
姐姐（Ⅳ_1_）	12.4	42.4	3.71	96	91	93	89	116	92
先证者（Ⅳ_2_）	52.2	108.3	3.06	99	89	94	92	2	4

参考值范围	12.5~14.5	29.0~43.0	2.00~4.00	86~116	86~120	78~128	80~120	86~114	70~140

**注** PT：凝血酶原时间；APTT：活化部分凝血活酶时间；FIB：纤维蛋白原；FⅡ∶C：凝血因子Ⅱ活性；FⅦ∶C：凝血因子Ⅶ活性；FⅧ∶C：凝血因子Ⅷ活性；FⅩ∶C：凝血因子Ⅹ活性；FⅤ∶C：凝血因子Ⅴ活性；FⅤ∶Ag：凝血因子Ⅴ抗原

2. 凝血活酶生成试验结果：表2及图2显示了先证者及其父亲的凝血活酶生成试验具体参数和曲线。与健康对照组相比，先证者及其父亲ETP和Peak均降低，ttPeak延长，延迟时间稍有延长。但先证者凝血活酶生成能力受损更为严重，ETP只有健康对照组的一半左右。

**表2:** 遗传性凝血因子Ⅴ缺陷症先证者及其父亲凝血活酶生成试验结果

受试者	延迟时间（min）	内源性凝血活酶生成潜力（nmol·min）	峰高（nmol）	到达峰值时间（min）
先证者	3.0	620	138.2	5.6
父亲	2.8	984	250.4	4.6
正常对照	2.6	1 320	385.7	4.3

**图2:**
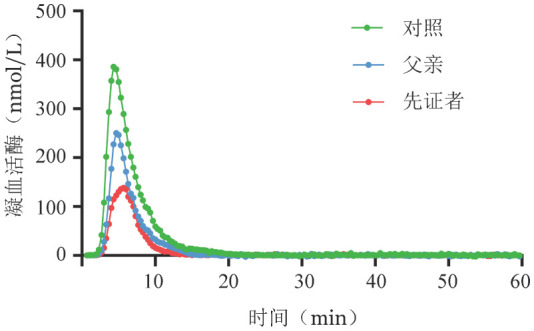
遗传性凝血因子Ⅴ缺陷症先证者及其父亲凝血活酶生成曲线

3. 先证者及其家系成员基因突变检测结果分析：Sanger测序显示先证者F5基因第15号外显子存在c.5128T>C纯合错义突变，导致第1682位色氨酸突变为精氨酸，即为p.Trp1682Arg，该突变已通过反向测序证实（图3）。先证者外祖父、父亲、母亲为p.Trp1682Arg杂合子。在健康对照组中未检出p.Trp1682Arg突变，排除了基因多态性可能。

**图3:**
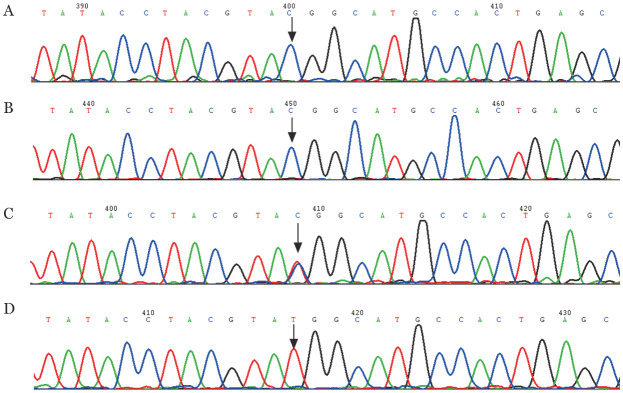
F5基因第15号外显子测序结果（箭头所示为突变位点） **A** c.5128T>C纯合突变型正向测序；**B** c.5128T>C纯合突变型反向测序；**C** c.5128T>C杂合突变型；**D** 野生型

根据美国医学遗传学与基因组学学会（ACMG）指南[7]判定c.5128T>C（p.Trp1682Arg）属于可能致病突变（PM2+PM3+PP1+PP3+PP4），证据如下：①该突变在多个正常对照人群数据库（gnomAD、千人数据库）中没有报道，属于罕见突变（PM2）；②在隐性遗传病中，在反式位置上检测到该突变位点（PM3）；③在该家系中，先证者父亲、母亲均检测到此位点突变（PP1）；保守性分析说明p.Trp1682在8个同源物种中为完全保守位点，5个在线生物信息学软件Mutation taster、SIFT、REVEL、PolyPhen-2、CADD预测可能有害（PP3）；④先证者PT和APTT明显延长，且FⅤ∶C和FⅤ∶Ag严重降低，家族成员基因表型也符合遗传性FⅤ缺陷症的家族史（PP4）。

4. 突变氨基酸保守性及生物信息学分析：Clustal X-2.1-win软件保守性分析结果提示p.Trp1682在8种同源物种中呈完全保守（图4）。5个在线软件对p.Trp1682的预测结果一致，提示该突变为一种致病突变。

**图4:**
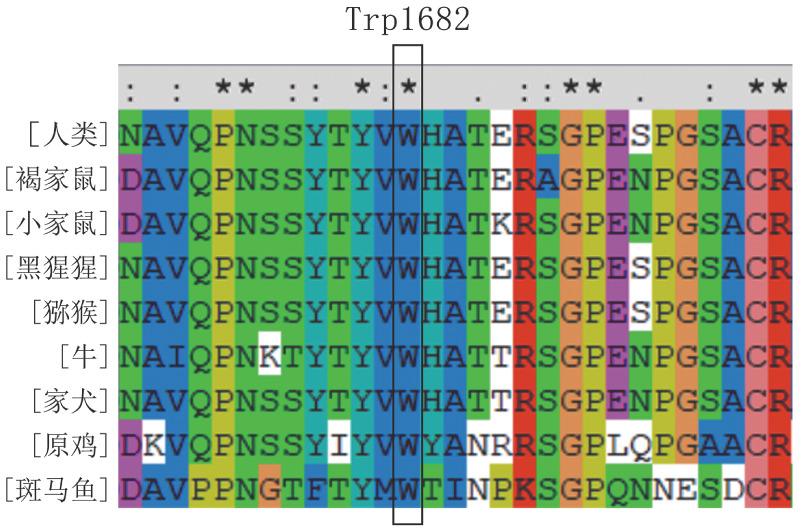
F5基因p.Trp1682在同源物种中比对结果（^★^、∶、·分别表示该氨基酸位点完全保守、高度保守、弱保守）

5. 蛋白模型分析：如图5所示，野生型FⅤ蛋白第1 682位Trp是一种具有双核环状结构的芳香族化合物，其芳香族侧链与第1 653位Ser形成一个氢键，并且Trp其他侧链与1 628位的Ile也形成了两个氢键。这三个氢键在蛋白质结构中共同参与构成了疏水核心，通过疏水效应促使蛋白质折叠，稳定蛋白质的立体结构。发生突变后，第1 682位氨基酸由非极性疏水性的Trp变为极性带正负电荷的Arg，原来与Ser1653之间的氢键消失，使FⅤ蛋白局部结构改变。

**图5:**
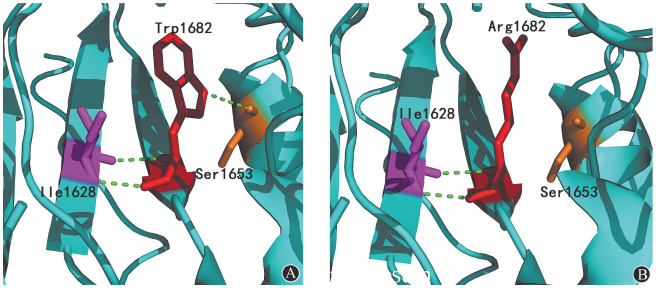
p.Trp1682Arg蛋白分子模型分析图（绿色虚线代表氢键） **A** 野生型凝血因子Ⅴ蛋白分子模型；**B** 突变型凝血因子Ⅴ蛋白分子模型

## 讨论

成熟FⅤ蛋白分为6个结构/功能区：从N端到C端依次是A1-A2-B-A3-C1-C2[5]。成熟FⅤ蛋白在促凝和抗凝过程中均起到了关键作用。当Arg^709^、Arg^1018^、Arg^1545^被水解，B结构域去除，此时FⅤ转化为活化的FⅤ（FⅤa）[8]。在凝血过程中，FⅤa与活化的FⅩ（FⅩa）、Ca^2+^、磷脂形成凝血酶原酶激活物共同催化凝血酶原转化为凝血酶[3]。此外，活化蛋白C（APC）可裂解FⅤa重链上Arg^306^、Arg^506^、Arg^679^使FⅤa失活[9]。失活的FⅤa辅助APC灭活凝血因子Ⅷ（FⅧ），从而参与抗凝过程[10]。FⅤ严重缺乏的患者通常是纯合或者复合杂合突变[2]。其常见的临床表现包括鼻出血、瘀斑、齿龈出血、轻微外伤后出血难止、月经过多等，中枢神经系统出血、消化道出血较为少见[3],[11]。但出血严重程度有时与FⅤ∶C关联性较差，FⅤ∶C 50％的患者仍表现各种出血症状[1]。

本家系先证者因无明显诱因双侧鼻出血至医院就诊，凝血表型结果PT和APTT显著延长，进一步检测发现FⅤ∶C和FⅤ∶Ag均低于5％，诊断为Ⅰ型FⅤ缺陷症。先证者父亲、母亲、外祖父的PT和APTT稍高于参考值范围上限，FⅤ∶C和FⅤ∶Ag为正常值的一半左右。基因测序结果显示：先证者F5基因第15外显子上存在c.5128T>C（p.Trp1682Arg）纯合错义突变，其父亲、母亲、外祖父携带p.Trp1682Arg杂合突变。该基因表型符合常染色体隐性遗传病的家族史，突变基因源自姑表近亲结婚的父母并遗传给女儿。常规凝血检查并不能完全反映体内真实的凝血环境，而凝血活酶生成试验可以通过连续测量凝血活酶浓度的变化，绘制其生成曲线，进一步确定血浆FⅤ的促凝能力[5],[12]。凝血活酶生成试验显示，先证者及其父亲的凝血活酶生成量及ETP与健康对照者相比均降低，尤其是先证者的凝血活酶生成能力受损更严重，ETP仅有健康对照者的一半左右；而二者延迟时间则没有明显差别，表明凝血活酶生成的起始时间较正常人没有明显变化。二者临床表现与凝血活酶生成试验结果一致：先证者表现为自发性鼻出血，而其父亲没有自发性出血症状。

人类基因组突变数据库（HGMD，https://www.hgmd.cf.ac.uk/ac/all.php）已收录200多种F5基因突变，其中包括错义突变、无义突变、剪接突变、移码突变[13]–[14]。其中大部分是杂合子突变，纯合子突变多见于近亲结婚家系[5]。本家系先证者为p.Trp1682Arg纯合突变，而其近亲结婚的父母均为p.Trp1682Arg杂合突变，正是近亲婚姻导致相同的隐性基因遗传给了后代，使其表现为纯合型FⅤ缺陷症。

新的突变位点p.Trp1682Arg位于FⅤ的A3结构域，保守性分析显示其在8个同源物种中高度保守，说明该位点在FⅤ的结构及功能中起到重要作用。FⅤa轻链通过A3结构域与FⅩa的EGF结构域相互作用共同参与了凝血酶原的组装[15]。据报道，一种识别FⅤa蛋白p.Asp1537-p.Lys1752区域的单克隆抗体可显著降低FⅤa与FⅩa结合和凝血酶原的生成[16]。此外，Steen等[17]构建了FⅤ蛋白p.His1683Glu突变位点，进一步的数据揭示了p.His1683周围区域是FⅩa结合位点并且该突变减少了凝血酶的形成，说明A3结构域也参与了与FⅩa的直接结合。而本文中先证者突变位点p.Trp1682与p.His1683临近，推测p.Trp1682Arg会影响FⅤa与FⅩa的结合从而使凝血酶原的生成降低，凝血活酶生成试验试验结果也验证了这一点，先证者的ETP仅有健康对照者的一半左右。对比突变前后蛋白模型发现，发生突变后，第1 682位氨基酸由非极性疏水性的Trp变为极性带正负电荷的Arg，突变后一个苯环丢失以及与Ser1653之间的氢键消失，可能会影响蛋白正常局部折叠，从而影响FⅤ的三级结构。这一改变可能影响FⅤa重链与轻链之间的相互作用，最终引起血浆FⅤ水平降低。有文献报道了其他位于A3结构域的p.Glu1608Lys突变[18]，该位点的体外表达试验表明，突变蛋白在mRNA水平正常表达，但在蛋白质的合成和分泌过程中受损，导致FⅤ水平降低。由于p.Trp1682位于p.Glu1608附近，推测p.Trp1682Arg造成的FⅤ水平降低机制可能与p.Glu1608Lys相同。

综上所述，本研究在一个遗传性FⅤ缺陷症家系中发现了一个国际上首次报道的p.Trp1682Arg错义突变，并初步探讨了可能的致病机制。p.Trp1682Arg错义突变可能是该家系FⅤ水平降低的主要原因，但具体的致病机制尚需的体外表达研究加以验证。
